# Functional proteomics of failed filtering blebs

**Published:** 2009-12-15

**Authors:** Takashi Kanamoto, Nazariy Souchelnytskyi, Yoshiaki Kiuchi

**Affiliations:** 1 Department of Ophthalmology and Visual Science, Graduate School of Biomedical Sciences, Hiroshima University, Hiroshima, Japan; 2 Karolinska Biomics Centre, Department of Oncology Pathology, Karolinska Institute, Stockholm, Sweden

## Abstract

**Purpose:**

To identify and determine the function of the proteins associated with failed filtering blebs following trabeculectomy.

**Methods:**

Tenon's tissues, obtained during surgery for failed filtering blebs or obtained during cataract surgery on normal eyes, were analyzed by proteomics. The proteins showing significant differences between the two tissues were selected for identification by mass spectrometry. The location and expression pattern of ribosomal S6 kinase 2 (RSK2), one of the altered proteins, were determined. The effect of basic fibroblast growth factor (bFGF) on the expression pattern and function of RSK2 in NIH3T3 fibroblast cells was then investigated by an RNA knockdown technique.

**Results:**

Eight proteins were found differentially expressed in failed filtering blebs; the identified proteins included those associated with intracellular signaling pathways. The expression of RSK2, one of the identified proteins, was found to be decreased compared with that of the control. RSK2 was located in Tenon’s tissue using an immunohistochemical technique. In culture, the bFGF-induced cell proliferation was inhibited by the RNA knockdown of *RSK2*. The level of mRNA and protein expression of actin was increased by *RSK2* RNA knockdown, but bFGF-induced protein expression of actin was not promoted by *RSK2* RNA knockdown. Whereas *RSK2* RNA knockdown increased the expression and activity of mitogen-activated protein kinase (*MAPK*), activation of MAPK induced by bFGF was not promoted by *RSK2* knockdown.

**Conclusions:**

The expression of eight proteins in the failed filtering blebs was significantly different from that in the Tenon’s capsules used as a control. The effect of *RSK2* expression on fibroblast cells suggests that *RSK2* may be associated with wound healing in filtering blebs.

## Introduction

Untreated ocular hypertension can lead to visual field defects [1], and one method of treating the condition is trabecular filtration surgery [2]. The objective of filtrating surgery is to create a functioning filtering bleb that can reduce the intraocular pressure (IOP). To achieve this, it is essential that the filtering bleb remains functional. However, the wound healing process after filtration surgery can be a significant negative factor in maintaining a functional bleb [3]. Filtrating surgery, which involves breaking tissue barriers and upsetting tissue homeostasis, is naturally incompatible with wound healing, which is the normal biological reaction to tissue damage. In fact, patients are often unable to obtain a permanent filtering effect after surgery due to successful wound healing [4].

Antimetabolic drugs, such as mitomycin-C, are used to inhibit wound healing during the early postoperative period [5], and they are therefore frequently adopted as concomitant therapy for filtrating surgery [6]. However, because cell death is not actively induced, despite the inhibition of fibroblast proliferation there may be cases in which a permanent effect is not attained [7]. Furthermore, the targeted cells are nonspecific, thus leading to some related side effects of toxicity to corneal and scleral cells [8,9]. Therefore, the method by which wound healing in the filtering bleb is controlled is a significant factor in ensuring good postoperative results.

Fibroblasts play a significant role in the process of wound healing [10]. In the first stage of healing, the damaged site is covered by fibroblasts. The fibroblasts repeatedly divide and proliferate in order to reach the required number of cells. The components for protecting the damaged site are then secreted in large amounts. These secreted materials include  components of extracellular matrix, such as collagen and fibronectin [11]. The wound healing process coats the sclerotomy site, where a scleral flap has been created, with the proliferated fibroblasts [12].

In the second stage of healing, the proliferated fibroblasts gradually begin to differentiate; this process is suspected to be mediated by various factors: transforming growth factor (TGF)-beta [13], connective-tissue growth factor (CTGF) [14], Rho-associated serine-threonine kinase (ROCK1) [15], and the matrix-metaloproteinases (MMPs) [16]. Unlike undifferentiated fibroblasts, the newly-differentiated myofibroblasts transform the secreted extracellular matrix into an actin-based component which creates stronger scar tissue [17]. Although there are several other therapies that have been attempted as concomitant therapy for secondary wound healing, none of these therapies has shown sufficient efficacy. This has hitherto been a major limitation of filtrating surgery [18-20].

To find a new way to inhibit wound healing (as an alternative to the currently available concomitant therapy), it is necessary to examine the proteins involved in the healing process of filtration blebs in more detail. To do this we collected samples of fibrous Tenon’s tissue of the scleral flap from patients who required bleb-revision surgery after filtration surgery and identified the protein families whose expressions were changed relative to those of control samples. We found that RSK2, which was one of the identified proteins, may be involved in the wound healing attributes of a filtering bleb.

## Methods

### Tissue samples and cells

All experiments were performed in accordance with the Association for Research in Vision and Ophthalmology’s statement on the use of animals in ophthalmic research. The protocol was approved by the Institutional Review Board of Hiroshima University.

Samples of Tenon’s capsule tissue covering the scleral flap of failed filtering blebs were collected during bleb revision surgery from three patients: a 58-year-old male, a 65-year-old male, and an 87-year-old female. Tenon’s capsule tissue samples were also collected from three patients undergoing cataract surgery, to be used as controls: a 58-year-old female, an 80-year-old male, and an 89-year-old female. NIH3T3 cells from mouse embryo fibroblasts were obtained from the American Type Culture Collection (ATCC; Manassas,VA). The cells were maintained in Dulbecco’s modified Eagle’s medium (DMEM; Sigma-Aldorich, St.Louis, MO) with 10% fetal bovine serum (FBS), penicillin, and streptomycin and incubated at 37° C with 5% CO_2_.

### Proteomics

Sample preparation, two-dimensional electrophoresis, and protein identification were performed as described in detail elsewhere [21]. In brief, the specimens were carefully washed in phosphate-buffered saline (PBS) and solubilized in sample buffer (8 M urea, 4% CHAPS, 0.5% dithiothreitol [DTT], IPG buffer, pH 3–10). The protein concentration was measured by the Bradford assay.

Samples, including 50 µg protein, were treated by a rehydration technique. Isoelectrofocusing, or first-dimension electrophoresis, was performed on strips with an immobilized pH gradient (pH 3–10 non-linear gradient, 18 cm; GE Healthcare, Buckinghamshire, UK) using IPGphor (GE Healthcare) according to manufacturer’s instructions. After the isoelectrofocusing, the strips were placed in equilibration buffer-1 (50 mM Tris-HCl, pH 8.8, 6.0 M urea, 2.0% SDS, 30% glycerol, 1% DTT), and then in equilibration buffer-2 (50 mM Tris-HCl, pH 8.8, 6.0 M urea, 2.0% SDS, 30% glycerol, 4% iodoacetamide). The equilibrated strips were loaded onto SDS-PAGE gel containing 12% polyacrylamide gel, and SDS-polyacrylamide gel electrophoresis (PAGE) was performed as second-dimension electrophoresis. After electrophoresis the gels were fixed in 7.5% acetic acid and 20% methanol, and sensitized in 25% ethanol, 0.2% sodium thiosulfate, and 3.4% sodium acetate. The gels were then stained with 0.25% silver nitrate and developed with 2.5% sodium carbonate and 0.04% formaldehyde. The silver-stained gels were scanned on an image scanner (EPSON, Tokyo, Japan) and the volume of the spots was determined with PD-Quest software (Bio-Rad Laboratories, Hercules, CA) following the manufacturer’s instructions The software included the equipment to correct and standardize automatically the difference of total staining of compared gels. Three gels from either failed filtering bleb or control samples were prepared independently and master gels were generated**.** The values of the volume of each matched protein spot on the master gels were compared. Spots with differences in expression were then identified by mass spectrometry.

The excited protein-containing spots were destained in 30 mM potassium ferricyanide and 100 mM sodium thiosulfate, and the gel samples were treated with 0.1 M sodium hydrocarbonate and washed with acetonitrile. After drying, in-gel digestion was performed with trypsin. Then, 0.1% trifluoroacetic acid (TFA) and 10% acetonitrile in water were used to extract the peptides, and the extract was desalted on a nano-column. After washing the column with 0.1% TFA in water, the matrix was eluted with acetnitrile containing alpha-cyano-4-hydroxycinnamic acid directly onto the MALDI target.

Spectra were generated by the MALDI-TOF-MS (Bruker Daltonics, Billerica, MA) spectrometer. The spectra were internally calibrated using known internal tryptic autodigestion peptides and searches were made in the NCBI database using ProFound software. For search criteria, tolerance was set on 0.5 Da, no restriction on pI, and species were set as “mammalian.”

### Reverse transcription-polymerase chain reaction (RT-PCR)

All of the RNA of the NIH3T3 cells was extracted using ISOGEN (Nippongene, Tokyo, Japan). After the DNAse treatment, 1 μg of the RNA samples was used to synthesize cDNA using the random primer (P6) with a PrimeScript 1st strand cDNA synthesis kit (TAKARA, Tokyo, Japan) following the manufacturer’s instructions. Then, PCRs were performed for *α-actin* between the sense primer 5'-AGG GAG TAA TGG TTG GAA TGG G-3' and the antisense primer 5'-CCA GGG AGG AAG AGG AGG CGG CCG TGG-3', and for glyceraldehyde 3-phosphate dehydrogenase gene (*GAPDH*) between the sense primer 5'-AAT GTG TCC GTC GTG GAT CT-3' and the antisense primer 5'-TCC ACC ACC CTG TTG CTG TA-3'. The PCR conditions for actin were initial denaturation at 95 ºC for 5 min, 35 cycles of 95 ºC for 1 min, 40 ºC for 1 min, and 72 ºC for 1 min; and a final extension at 72 ºC for 6 min. For GAPDH, the conditions were initial denaturation at 95 ºC for 5 min, 35 cycles of 95 ºC for 1 min, 56 ºC for 1 min, and 72 ºC for 1 min; and a final extension at 72 ºC for 6 min by using LA-Taq polymerase (TAKARA). Of the resulting products, 10μl was electrophoresed on a 1.5% agarose gel containing ethidiumbromide (0.3 μg/ml) for actin and GAPDH.

### Western blot analysis

Western blot analysis was performed as described in detail [22]. Lysates of the tissues in sample buffer for proteomics were directly subjected to the SDS-PAGE technique. The NIH3T3 cells were treated in lysis buffer (150 mM NaCl, 20 mM Tris-HCl pH 7.4, 1% Triton-X, 0.5% deoxychorate, 1 mM phenylmethylsulfonyl fluoride, 10 mM NaF, 20 mg/ml aprotinin) and the lysates were subjected to SDS-PAGE. The proteins were transferred onto a nitrocellulose membrane, Hybond-C (GE Healthcare), blocked with 5% milk in TBS-T buffer (10 mM Tris-HCl pH 7.4, 150 mM NaCl, 0.1% Tween-20) , and incubated with a goat polyclonal antibody for RSK2, an anti-actin antibody, an anti-phospho MAPK/ERK antibody (SantaCruz), an anti-non-phosphorylayted MAPK/ERK antibody (Sigma-Aldorich), or an anti-αTubullin antibody (SantaCruz). Horse radish peroxide-conjugated secondary antibodies were used, and the blots were developed with  enhanced chemiluminescence (ECL).

### Immunohistochemistry

Tissue samples were washed with PBS three times and fixed with 4% paraformaldehyde. The fixed samples were treated with 12%, 15%, and 18% sucrose. The samples were sectioned on a cryostat, and the sections were incubated with mouse monoclonal anti-human RSK2 antibody (E-1: Santacruz Biotechnology, Santa Cruz, CA). The slides were then incubated with fluorescein isothiocyanate (FITC)-conjugated secondary antibody (Sigma-Aldorich) and mounted in Fluoromount G (SouthernBiotech, Birmingham, AL). The sections were examined with a confocal laser microscope (BZ-8100: Keyence Corp., Osaka, Japan).

### Cell biological assays

Mouse RSK2 siRNA (Santacruz Biotechnology) was transfected into NIH3T3 cells using the RNAi MAX system (Invitrogen Corp., Carlsbad, CA), according to the manufacturer’s instructions. After culturing for 24 h the cells were treated with recombinant basic fibroblast growth factor (bFGF: Sigma-Aldrich) for 24 h. The number of living cells was determined by the MTS assay which was performed with Cell Titer 96 AQUEOUS One Solution Reagent (Promega, Madison, WI). The products of the reaction were identified by spectral analysis profiles and the results were statistically analyzed.

## Results

### Two-dimensional proteome maps of tissue in failed filtering blebs

**Figure 1:**
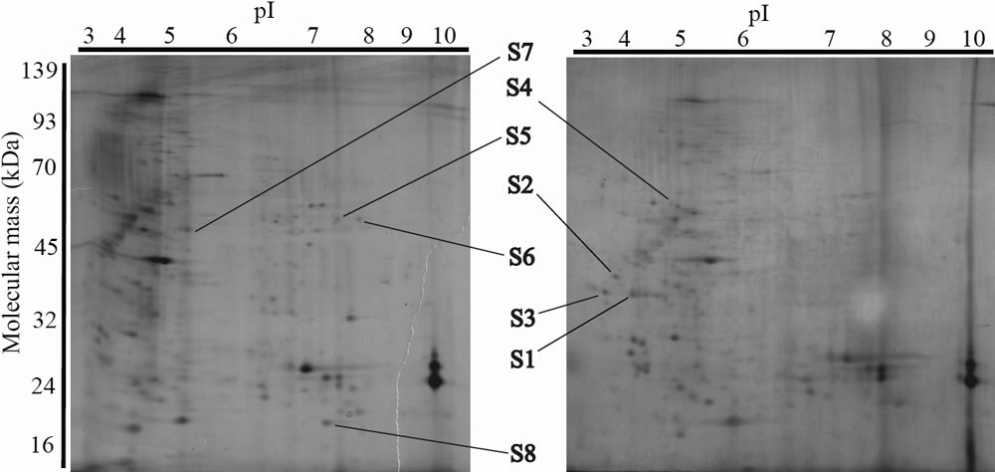
Images of two-dimensional electrophoresis gels with annotation indicating the spots of the identified proteins. The image on the left shows the silver-stained gel of a failed filtering bleb. The right gel shows the results for a control tissue. Spots S1 through S8 are indicated. The pI gradient of first dimension electrophoresis is shown above the gels, and the migration of the molecular mass markers for SDS-PAGE in the second dimension is shown at the side. Gel images shown are representative.

To identify the proteins whose expression was up- or downregulated in the failed filtering blebs, we compared the proteome of Tenon tissue from failed filtering blebs to those from the control Tenon’s capsule. The total tissue lysates were resolved by two-dimensional gel electrophoresis. We detected approximately five hundred protein spots on the two-dimensional gels after silver staining (Figure 1). We analyzed three gels for each of the samples to ensure the reliability of the spotting as an indicator of significant change, and a master gel was constructed for each sample using PD-Quest software.

The volume of all of the protein spots was quantified, and the maximum volume of one spot was 24,806 units. Small and weak spots whose protein volume was less than 3000 units were eliminated from analysis. Weak spots are thought likely to fail protein identification by mass spectrometry in the next experimental step. This left 96 protein spots from the failed filtering bleb and 88 from the control. Of these, matched spots whose volume was expressed equally in both the failed filtering bleb and the control were also eliminated; this left 23 protein spots from the failed filtering bleb and 16 from the control. The remaining gels were carefully examined to confirm whether spots were present or absent. Finally, eight spots that revealed a significant increase or decrease in volume were selected, and MALDI TOF mass spectrometry was performed to identify their proteins. All eight spots were successfully identified, with high quality spectra and sufficient reliability.

Four of the eight (50%) proteins, S1, S2, S3, and S4, were not expressed in the failed filtering bleb and the others, S5, S6, S7, and S8, were specifically identified in the failed filtering bleb. We also identified POR in two protein spots, S2 and S3 (Table 1).

**Table 1:** Differentially expressed proteins identified by proteomics from Tenon tissue obtained from failed filtering bleb.

**Spot­**	**Protein**	**Sequence**				**Theoretical value**		**Experimental value**	
		**Probability**	**Est'd Z**	**Coverage (%)**	**ncbi ID**	**pH**	**Mr (kDa)**	**pH**	**Mr (kDa)**
**Spots identified only in control tissue**								
S1	ribosomal protein S6 kinase 3	1.0e+000	1.43	21	AAC82495.1	6.1	66.28	4.5	42
S2	POR2	1.0e+000	1.72	27	AAG33132.1	7.2	61.89	3.5	42
S3	POR2	1.0e+000	1.2	21	AAG33132.1	7.2	61.89	4.0	42
S4	GalNAc alpha-2,6-sianyltransferase 1	1.0e+000	0.85	18	NP_060884	10.1	68.55	5.0	60
**Spots identified only in the failed filtering bleb**								
S5	Fibrin-beta	1.0e+000	1.67	32	0401173A	8.3	51.53	8.0	55
S6	PSAPL1 protein	5.3e+001	0.3	7	AAH68579.1	8.7	60.74	8.5	55
S7	Hypothetical protein	9.8e-001	1.42	32	CAD38695.1	10.1	68.73	6.0	50
S8	1-aminocyclopropane1-carboxylate synthetase	1.0e+000	2.43	22	NP_115981.1	6.0	57.88	8.5	35

S1 to S8 represent ID number of spots and sequence coverage, and the theoretical value of pI and Mr were obtained from the Pro Found search. The calculations of the experimental pI and Mr were based on the migration of the protein on a 2D gel.

### Downregulation of RSK2 protein expression in the failed filtering bleb

**Figure 2:**
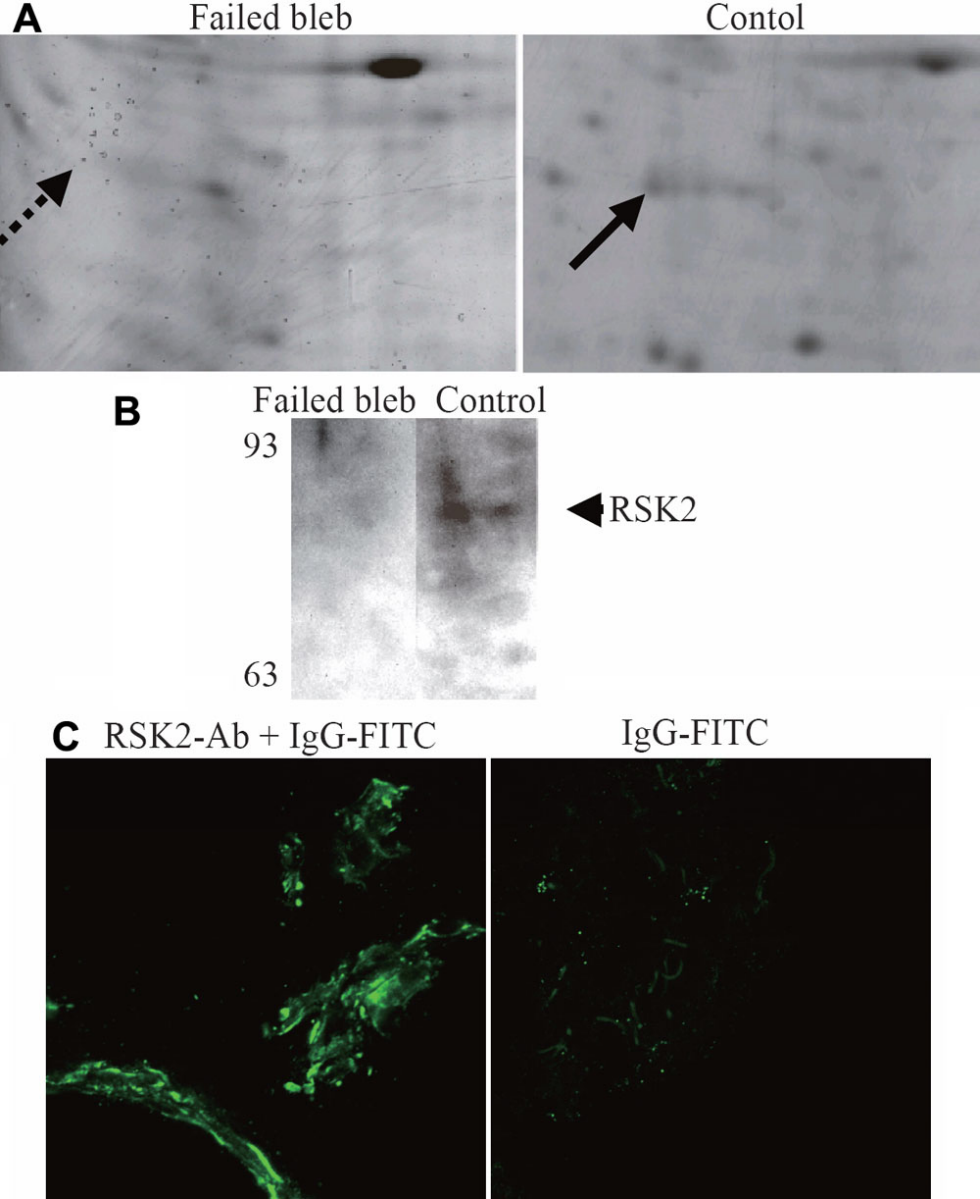
This shows changes in expression of RSK2; a portion of 2D-gel that includes the protein spot of RSK2 (arrow) is shown at high magnification. **A**: Total lysates of tissue sample from failed filtering blebs (Failed bleb) and from Tenon obtained during cataract surgery (Control) were applied to 12% polyacrylamide gel (SDS PAGE), and blotted with anti-RSK2 antibody. Migration position of RSK2 is indicated. **B**: The panel on the left shows RSK2 expression in Tenon’s capsule derived from cataract surgery stained with an anti-RSK2 antibody. The right-hand panel is stained without the primary antibody, as a negative control. Arrows show the actual protein spots, identified as RSK2.

Among the identified proteins, we focused on RSK2 (S1) whose expression was significantly downregulated in the failed filtering blebs. (Figure 2A) To confirm the results of proteomics, western blot analysis was performed to determine the level of expression of the RSK2 protein. This indicated that expression of RSK2 was lower in the failed filtering blebs than in the Tenon’s capsule obtained from the controls (Figure 2B). To confirm expression of RSK2 in the Tenon’s tissue, immunostaining was also performed on the Tenon’s tissue obtained from the controls. Our results showed that RSK2 was expressed in the control Tenon’s tissue (Figure 2C).

### Effect of RSK2 RNA knockdown on fibroblasts in vitro

**Figure 3:**
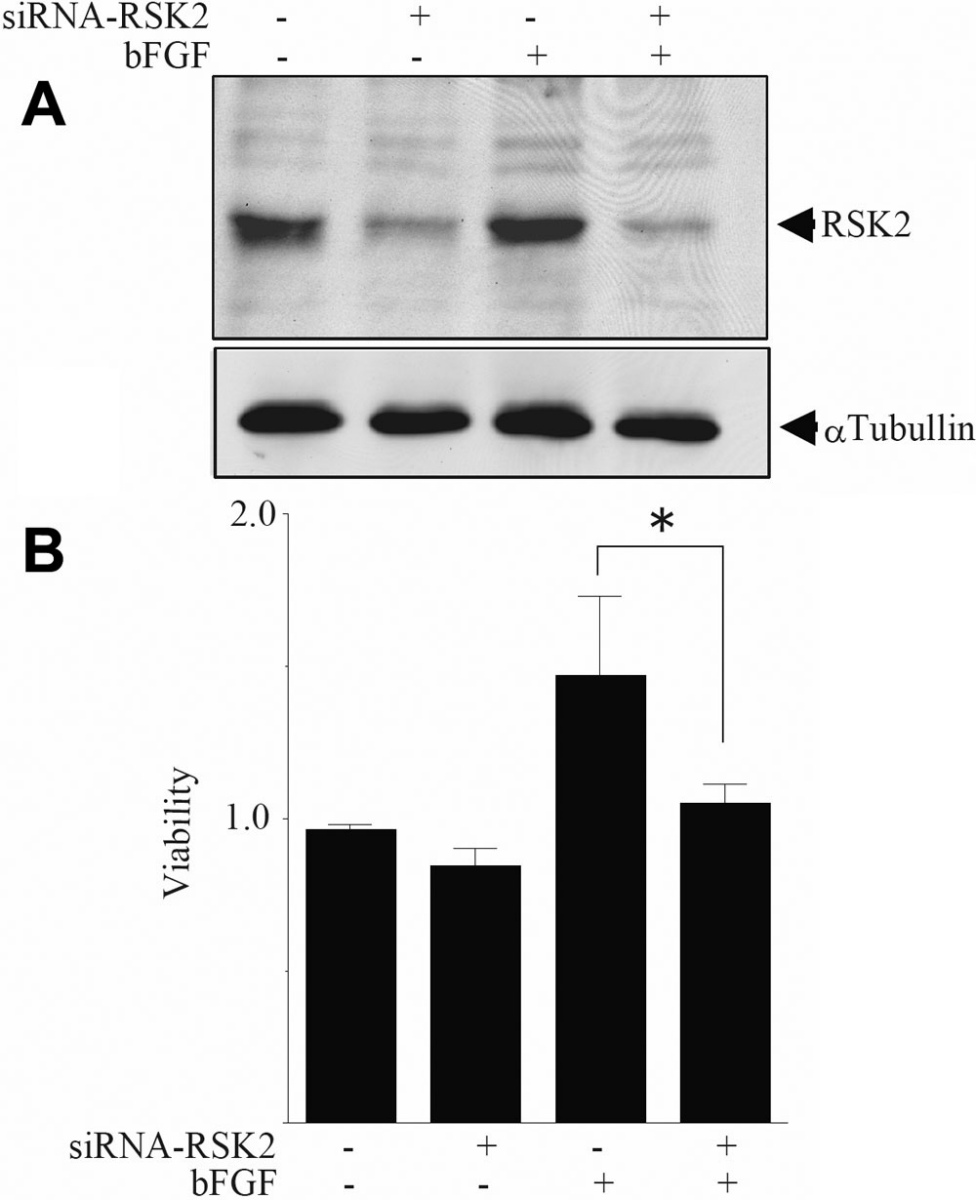
This shows the effect of *RSK2* siRNA on proliferation of fibroblasts. **A**: NIH3T3 cells were transfected with *RSK2* siRNAs and cultured for 24 h. After that, cells were treated with or without 0.33 μg/ml bFGF for 24 h. Cells were lysed and the whole cell extract was loaded onto 7.0% polyacrylamide gel (SDS-PAGE). Endogenous RSK2 expression was blotted with an anti-RSK2 antibody as indicated. Blots with an anti-α tubulin antibody were also performed to standardize the total protein volume loaded. **B**: The number of living cells was estimated by MTS assays and three independent experiments were performed. The results were statistically analyzed and the ratio of surviving cells (viable) is shown as the mean±standard error of the means. (The asterisk indicates a p<0.05, Student’s *t* test).

To further investigate the effects of RSK2, an RNA knockdown of *RSK2* was performed on cultured NIH3T3 cells. The cells were transfected with the siRNAs of *RSK2* and western blot analysis showed that the endogenous expression of *RSK2* was significantly decreased (Figure 3A). These results confirm that the process of RNA knockdown of *RSK2* acts on the NIH3T3 cells.

To determine what role *RSK2* plays in the proliferation of fibroblasts, NIH3T3 cells were exposed to bFGF, a known promoter of fibroblast proliferation [23], in the presence or absence of *RSK2* RNA knockdown. In the absence of bFGF, the ratio of the living NIH3T3 cells with the RNA knockdown was 0.842±0.057 (average±standard error of mean) which was not significantly different from that in non-treated cells at 0.96±0.018. The addition of bFGF significantly increased the number of living NIH3T3 cells to 1.468±0.263, and the simultaneous presence of *RSK2* RNA knockdown significantly decreased the number of living cells to 1.053±0.061 (Figure 3B). These results indicate that *RSK2* RNA knockdown inhibited the proliferation of fibroblasts induced by bFGF, and thus we believe that *RSK2* may be associated with wound healing.

### Effect of RSK2 on actin expression, as a marker of wound healing and MAPK activity

**Figure 4:**
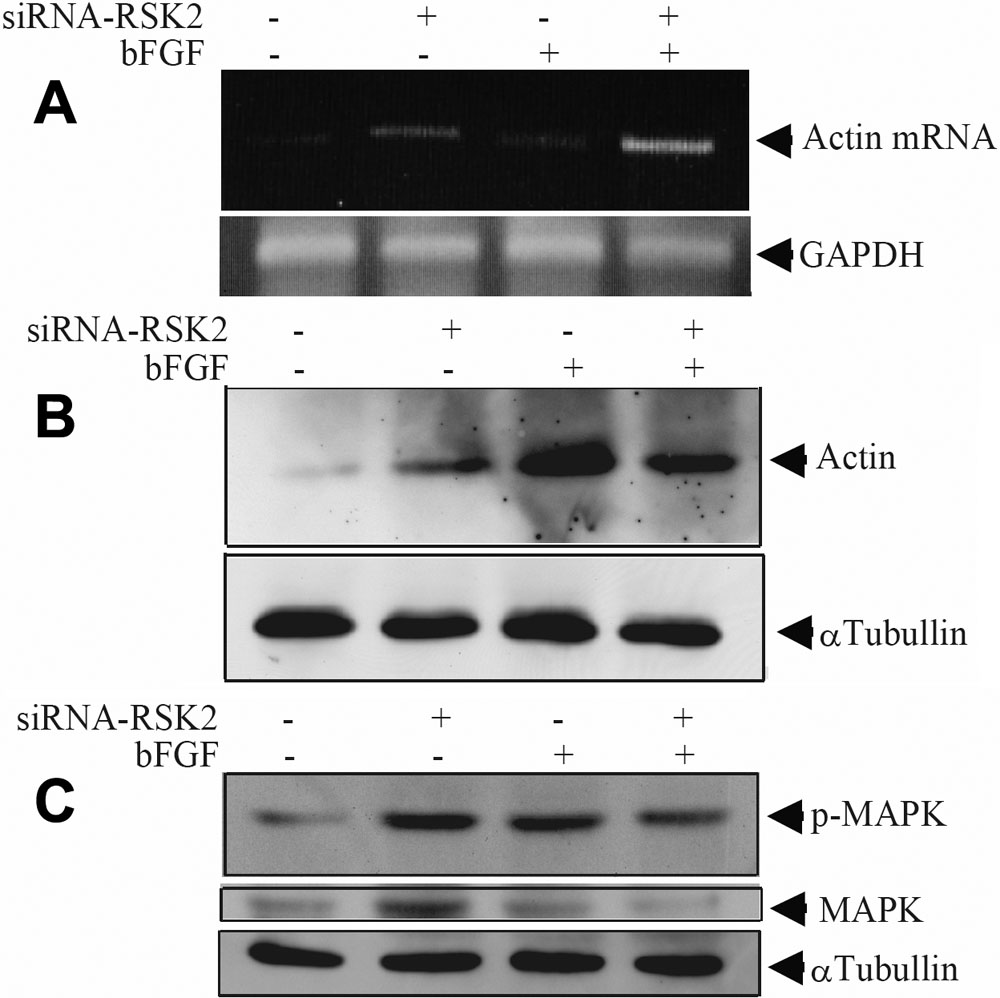
These images show the effect of *RSK2* siRNA on the expression of *actin* and *MAPK*. **A:** siRNAs of *RSK2* were transfected into NIH3T3 cells. Twenty-four hours later, cells were treated with or without 0.33 μg/ml bFGF for 24 h. RT-PCR analysis was performed for *actin* mRNA. The upper panel shows PCR product of *actin* that were separated by electrophoresis in 1.5% agarose gel. The lower panel shows the PCR products of *GAPDH* as an internal control. **B**: The whole cell extract was applied to 7.0% polyacrylamide gel (SDS-PAGE) and blotted with anti-actin antibody (upper panel). Blot with anti-α tubulin antibody shows the standardized loaded volume (lower panel). **C**: The whole cell extract was also applied to 8.0% polyacrylamide gel (SDS-PAGE) and blotted with anti-phosphorylated MAPK (p-MAPK) antibody (upper panel) and anti-non phosphorylated MAPK antibody (middle panel).

To investigate the molecular function of RSK2, we observed the expression of actin and MAPK (mitogen-activated protein kinase) in the absence or presence of bFGF. *RSK2* RNA knockdown promoted both mRNA and protein expression of actin in the absence of bFGF (Figure 4A,B). Although treatment with bFGF also induced the protein expression of actin, this upregulation of actin expression was not promoted by *RSK2* RNA knockdown (Figure 4B). Expression of MAPK, however, was promoted by *RSK2* RNA knockdown and MAPK activity was also up-regulated in the absence of bFGF. The MAPK activity induced by treatment with bFGF was not promoted by *RSK2* RNA knockdown (Figure 4C). Thus, the expression of actin was regulated by *RSK2* via downregulation of MAPK, and we know that expression of actin is an important component of wound healing. This means that RSK2 may have an inhibitory effect on wound healing by acting on fibroblasts, and this supports our observation that RSK2 was downregulated in failed filtering blebs.

## Discussion

Although the ideal control for the failed filtering bleb is a specimen from the eye of a successful trabeculectomy, it is impossible, clinically and ethically, to collect samples from a functional filtering bleb. So, our investigation was limited physiologically, but samples derived from the results of proteomics showed several protein candidates that might contribute to the failure of filtering blebs. One of these, RSK2, is one of the three mammalian homologs of RSKs [24] and was downregulated in the failed filtering blebs. RSK2 inhibits the differentiation of fibroblast cells in the absence of bFGF; it is involved in the mitogen-activated protein kinase (MAPK) pathway and dissociates from MAPK following mitogen stimulation [25]. MAPK activates growth factors involved in the phosphorylation of the cyclic adenosine monophosphate-response element-binding protein (CREB), a critical regulator of early gene transcription [26]. Although an activated MAPK phosphorylates RSK2 [27], RSK2 itself reduces the expression and phosphorylation of MAPK. It is suspected that RSK2 plays a role for negative feedback mechanism for the activity of MAPK. Furthermore, actin is one of the main components responsible for wound healing of filtering blebs, and RSK2 phosphorylates actin-binding protein, ABP-280 [28], inducing the activation of RSK2. These findings suggest that it is the MAPK signaling, whose activity is reduced by RSK2, that promotes wound healing, and this is compatible with our finding that RSK2 expression was decreased in failed filtering blebs.

RSK2 promotes the activation of MAPK induced by bFGF and promoted the function of bFGF, cell proliferation, and differentiation of fibroblast cells. FGF is a ligand of the receptor for the MAPK signaling pathway and regulates the cell cycle. bFGF binds directly to RSK2 and activated RSK2 promotes the cell cycle from G_0_ phase to G_1_ phase [29]. Downregulation of *RSK2* inhibits the proliferation of fibroblasts in the filtering bleb and is suspected to be needed for the preparation of the post-stage of wound healing, the differentiation of fibroblasts to myofibroblasts. Furthermore, RSK2 has a multiple function in the process of transcription and MAD1 (MAX dimerization protein-1), which suppresses myc-mediated cell transformation, is one of the substrates [30]. RSK also phosphorylates filamin-A, a membrane-associated, cytoskeletal protein that crosslinks actin filaments [31]. Thus, RSK is also linked to biological processes in the process of post-translational modification and could have a role in the post-stage of wound healing.

ST6GAlNAc1 (GalNAc alpha-2, 6-sianyltransferase 1: S4) was also identified as one of the downregulated proteins in failed filtering blebs. ST6GAlNAc1 reduces the number of O-glycosylation sites on the cell surface glycoconjugate [32], and its activity is important for shaping the cell phenotype [33]. Another protein, POR2 (pyruvate oxidoreductase: S2/S3), which is a metabolic anaerobic enzyme that is reducted by metronidazole [34], was also downregulated in failed filtering blebs. The protein ACC synthetase (1-aminocyclopropane1-carboxylate synthetase: S8), one of the transcriptional modulators [35], was upregulated in the failed filtering bleb. These metabolic enzymes may play important roles during wound healing, but the exact process has still not been determined. The protein functions of PSAPL1 (S6) and hypothetical protein (S7) were not analyzed in this study. They should be investigated further to determine their role in failed filtering blebs.

In conclusion, proteome analyses identified eight proteins in failed filtering blebs whose level of expression was significantly different from that in control tissues. A functional analysis of RSK2 indicated that RSK2 may be associated with wound healing of filtering blebs.

## References

[1] Quigley HA Number of people with glaucoma worldwide. Br J Ophthalmol 1996 80 389 93 8695555 10.1136/bjo.80.5.389 PMC505485

[2] Migdal C Gregory W Hitchings R Long-term functional outcome after early surgery compared with laser and medicine in open-angle glaucoma. Ophthalmology 1994 101 1651 7 7936562 10.1016/s0161-6420(94)31120-1

[3] Cordeiro MF Chang L Lim KS Daniels JT Pleass RD Siriwardena D Khaw PT Modulating conjunctival wound healing. Eye 2000 14 536 47 11026984 10.1038/eye.2000.141

[4] Tahery MM Lee DA Review: pharmacologic control of wound healing in glaucoma filtration surgery. J Ocul Pharmacol 1989 5 155 79 2666533 10.1089/jop.1989.5.155

[5] Doyle JW Sherwood MB Khaw PT McGrory S Smith MF Intraoperative 5-fluorouracil for filtaration surgery in the rabbit. Invest Ophthalmol Vis Sci 1993 34 3313 9 8225866

[6] Singh J O’Brien C Chawla HB Success rate and complications of intraoperative 0.2 mg/ml mitomycin C in trabeculetomy surgery. Eye 1995 9 460 6 7498567 10.1038/eye.1995.107

[7] Daniels JT Occleston NL Crowston JG Khaw PT Effects of antimetabolite induced cellular growth arrest on fibroblast-fibroblast interactions. Exp Eye Res 1999 69 117 27 10375456 10.1006/exer.1999.0684

[8] Sacu S Rainer G Findl O Georgopoulpos M Vass C Correlation between the early morphological appearance of filtering blebs and outcome of trabeculectomy with mitomycin C. J Glaucoma 2003 12 430 5 14520152 10.1097/00061198-200310000-00006

[9] Shields MB Scroggs MW Sloop CM Simmons RB Clinical and histopathologic observations concerning hypotony after trabeculectomy with adjunctive mitomycin C. Am J Ophthalmol 1993 116 673 83 8250068 10.1016/s0002-9394(14)73465-8

[10] Grierson I Joseph J Miller M Day JE Wound repair: the fibroblast and the inhibition of scar formation. Eye 1988 2 135 48 3058520 10.1038/eye.1988.27

[11] Lama PJ Fechtner RD Antifibrotics and Wound healing in glaucoma surgery. Surv Ophthalmol 2003 48 314 46 12745005 10.1016/s0039-6257(03)00038-9

[12] Ignotz RA Massague J Transforming Growth Factor-β stimulates the expression of fibronectin and collagen and their incorporation into the extracellular matrix. J Biol Chem 1986 261 4337 45 3456347

[13] Wipff P-J Rifkin DB Meister J-J Hinz B Myofibroblast contraction activates latent TGF-β1 from the extracellular matrix. J Cell Biol 2007 179 1311 23 18086923 10.1083/jcb.200704042 PMC2140013

[14] Sherwood MB A sequential, multiple-treatment, targeted approach to reduce wound healing and failure of glaucoma filtration surgery in a rabbit model. Trans Am Ophthalmol Soc 2006 104 478 92 17471357 PMC1809919

[15] Meyer-ter-Vehn T Sieprath S Katzenberger B Gebhardt S Grehn F Schlunck G Contractility as a prerequisite for TGF-beta-induced myofibroblast transdifferentiation in human tenon fibroblasts. Invest Ophthalmol Vis Sci 2006 47 4895 904 17065504 10.1167/iovs.06-0118

[16] Chintala SK Wang N Diskin S Mattox C Kagemann L Fini ME Schumann JS Matrix metalloproteinase gelatinase B (MMP-9) is associated with leaking glaucoma filtering blebs. Exp Eye Res 2005 81 429 36 16185954 10.1016/j.exer.2005.03.001 PMC1941659

[17] Desmouliere A Geinoz A Gabbiani F Gabbiani G Transforming Growth Factor-β1 Induces α-Smooth Muscle Actin Expression in Granulation Tissue Myofibroblasts and in Quiescent and Growing Cultured Fibroblasts. J Cell Biol 1993 122 103 11 8314838 10.1083/jcb.122.1.103 PMC2119614

[18] Eibl KH Banas B Kook D Ohlmann AV Priglinger S Kampik A Welge-Luessen UC Alkylphosphocholines:A New Therapeutic Option in Glaucoma Filtration Surgery. Invest Ophthalmol Vis Sci 2004 45 2619 24 15277485 10.1167/iovs.03-1351

[19] Angella GJ Sherwood MB Balasubramanian L Doyle JW Smith MF Setten GV Goldstein M Schultz GS Enhanced short-term plasmid transfection of filtration surgery tissues. Invest Ophthalmol Vis Sci 2000 41 4158 62 11095609

[20] Einmahl S Behar-Cohen F D’Hermies F Rudaz S Tabatabay C Renard G Gurny R A new poly(ortho ester)-based drug delivery system as an sdjunct treatment in filtering surgery. Invest Ophthalmol Vis Sci 2001 42 695 700 11222529

[21] Kanamoto T Hellman U Heldin C-H Souchelnytskyi S Functional proteomics of transforming growth factor-beta1-stimulated Mv1Lu epithelial cells: Rad51 as a target of TGF beta1-dependent regulation of DNA repair. EMBO J 2002 21 1219 30 11867550 10.1093/emboj/21.5.1219 PMC125881

[22] Kanamoto T Mota M Takeda K Rubin LL Miyazono K Ichijo H Bazenet CE Role of apoptosis signal-regulating kinase in regulation of the c-Jun N-terminal kinase pathway and apoptosis in sympathetic neurons. Mol Cell Biol 2000 20 196 204 10594022 10.1128/mcb.20.1.196-204.2000 PMC85075

[23] Bailly K Soulet F Leroy D Amalric F Bouche G Uncoupling of cell proliferation and differentiation activities of basic fibroblast growth factor. FASEB J 2000 14 333 44 10657989

[24] Moller DE Xia CH Tang W Zhu AX Jakubowski M Human risk isoforms: cloning and characterization of tissue specific ezpression. Am J Physiol 1994 266 C351 9 8141249 10.1152/ajpcell.1994.266.2.C351

[25] Roux PP Richards SA Blenis J Phosphorylation of p90 ribosomal S6 kinase (RSK) regulates extracellular signal-regulated kinase docking and RSK activity. Mol Cell Biol 2003 23 4796 804 12832467 10.1128/MCB.23.14.4796-4804.2003 PMC162206

[26] Xing J Ginty DD Greenberg ME Coupling of the RAS-MAPK pathway to gene activation by RSK2, a growth factor-regulated CREB kinase. Science 1996 273 959 63 8688081 10.1126/science.273.5277.959

[27] Anjum R Blenis J The RSK family of kinases: emerging roles in cellular signaling. Nat Rev Mol Cell Biol 2008 9 747 58 18813292 10.1038/nrm2509

[28] Ohta Y Hartwig JH Phosphorylation of actin-binding protein 280 by growth factors is mediated by p90 ribosomal protein S6 kinase. J Biol Chem 1996 271 11858 64 8662682 10.1074/jbc.271.20.11858

[29] Soulet F Bailly K Roga S Lavigne A-C Amalric F Bouche G Exogenously added fibroblast growth factor 2 (FGF-2) to NIH3T3 cells interacts with nuclear ribosomal S6 kinase 2 (RSK2) in a cell cycle-dependent manner. J Biol Chem 2005 280 25604 10 15879597 10.1074/jbc.M500232200

[30] Zhu J Blenis J Yuan J Activation of PI3/Akt and MAPK pathways regulates myc-mediated transcription by phosphorylating and promoting the degradation of Mad1. Proc Natl Acad Sci USA 2008 105 6584 9 18451027 10.1073/pnas.0802785105 PMC2373325

[31] Woo MS Ohta Y Rabinovitz I Stossel TP Blenis J Ribosomal S6 kinase regulates phosphorylation of filamin A on an important regulatory sites. Mol Cell Biol 2004 24 3025 35 15024089 10.1128/MCB.24.7.3025-3035.2004 PMC371131

[32] Sewell R Backstrom M Dalziel M Gschmeissner S Karlsson H Noll T Gatgens J Clausen H Hansson GC Burchell J Taylor-Papadimitriou J The ST6GalNAc-l sialyltransferase localizes throughout the golgi and lis responsible for the synthesis of the tumor-associated sialyl-Tn 0-glycan in human breast canaer. J Biol Chem 2006 281 3586 94 16319059 10.1074/jbc.M511826200

[33] Donadio S Dubois C Fichant G Roybon L Guillemot J-C Breton C Ronin C Recognition of cell surface acceptors by two human α-2,6-sialyltransferases produced in CHO cells. Biochimie 2003 85 311 21 12770770 10.1016/s0300-9084(03)00080-4

[34] Muller J Sterk M Hemphill A Muller N Characterization of Giardia Iamblia WB C6 clones resistant to nitazoxanide and to metronidazole. J Antimicrob Chemother 2007 60 280 7 17561498 10.1093/jac/dkm205

[35] Kimura K Wakamatsu A Suzuki Y Ota T Nishikawa T Yamashita R Yamamoto J Sekine M Tsuritani K Wakaguri H Ishii S Sugiyama T Saito K Isono Y Irie R Kushida N Yoneyama T Otsuka R Kanda K Yokoi T Kondo H Wagatsuma M Murakawa K Ishida S Ishibashi T Takahashi-Fujii A Tanase T Nagai K Kikuchi H Nakai K Isogai T Sugano S Diversification of transcriptional modulation: Large-scale identification and characterization of putative alternative promoters of human genes. Genome Res 2006 16 55 65 16344560 10.1101/gr.4039406 PMC1356129

